# Data from the Indian drug regulator and from Clinical Trials Registry-India does not always match

**DOI:** 10.3389/fmed.2024.1346208

**Published:** 2024-02-15

**Authors:** Iqbal S. Bhalla, Adithi Gopadi Ravindranath, Ravi Vaswani, Gayatri Saberwal

**Affiliations:** ^1^School of Geography and the Environment, Oxford University, Oxford, United Kingdom; ^2^Department of Biotechnology, PES University, Bengaluru, India; ^3^Yenepoya Deemed to be University, Mangaluru, India; ^4^Institute of Bioinformatics and Applied Biotechnology, Bengaluru, India

**Keywords:** regulatory trials, Central Drugs Standard Control Organization, data integrity, trial registry metaresearch, data quality, Institutional Ethics Committees, transparency in clinical trials

## Abstract

**Introduction:**

In India, regulatory trials, which require the drug regulator’s permission, must be registered with the Clinical Trials Registry-India (CTRI) as of 19 March 2019. In this study, for about 300 trials, we aimed to identify the CTRI record that matched the trial for which the regulator had given permission. After identifying ‘true pairs’, our goal was to determine whether the sites and Principal Investigators mentioned in the permission letter were the same as those mentioned in the CTRI record.

**Methods:**

We developed a methodology to compare the regulator’s permission letters with CTRI records. We manually validated 151 true pairs by comparing the titles, the drug interventions, and the indications. We then examined discrepancies in their trial sites and Principal Investigators.

**Results:**

Our findings revealed substantial variations in the number and identity of sites and Principal Investigators between the permission letters and the CTRI records.

**Discussion:**

These discrepancies raise concerns about the accuracy and transparency of regulatory trials in India. We recommend easier data extraction from regulatory documents, cross-referencing regulatory documents and CTRI records, making public the changes to approval letters, and enforcing oversight by Institutional Ethics Committees for site additions or deletions. These steps will increase transparency around regulatory trials running in India.

## Introduction

In India, the manufacture, licensing, and importing of drugs, as well as the conduct of clinical trials, are regulated by the Central Drugs Standard Control Organization (CDSCO), headed by the Drug Controller General of India (DCGI). Hereafter we will usually refer to CDSCO and the DCGI as the regulator. Trials that require the permission of the regulator to run are termed ‘regulatory trials’. According to the New Drugs and Clinical Trial Rules, 2019 (hereafter referred to as the New Drug Rules), since 19 March 2019 such studies are required to be registered with Clinical Trials Registry-India (CTRI) (1). It is the primary sponsor’s responsibility to ensure that the trial is registered with CTRI. Also, since 1 April 2018, all registrations with CTRI have been required to be prospective, that is, before the first participant is enrolled (2). Globally this is a recommended practice (3) since prospective registration ensures that the sponsor does not have an opportunity to look at the results of the trial and alter the endpoints.

Trials registered with CTRI (4) are assigned CTRI numbers in the format CTRI/year/month/number (for example, CTRI/2020/06/026192). It is not mandatory to pre-register the study in CTRI before applying to the regulator for permission to run it, and perhaps for that reason the CTRI number is not available in the letter from the regulator that gives permission to run the trial. Likewise, although there is a field *Regulatory Clearance Status from DCGI* in the CTRI record, where the ‘status’ may be ‘Approved/Obtained’, there are no further details about the letter from the regulator. As such, it is not possible to easily and unambiguously link a given letter of approval from the regulator with the corresponding CTRI record.

In this study, for a set of trials, we aimed to identify the CTRI record that matched the trial for which the regulator had given permission. After identifying ‘true pairs’, our goal was to determine whether the sites and Principal Investigators (PIs) mentioned in the letter from the regulator were the same as those mentioned in the CTRI record. We anticipated that there may be some discrepancies. The rationale for expecting some discrepancies is as follows. Data in trial registries are prone to errors in the form of missing information, internally inconsistent information, discrepancies between data relating to the same trial in different registries, and discrepancies regarding a given trial in the data with the regulator, a trial registry and a publication. Such issues have been recorded for other registries as well, primarily ClinicalTrials.gov, of the United States, which is by far the biggest (5) and clearly the most studied of the public registries. In our own work, we have identified such issues in various fields of the CTRI records (6–8). Researchers around the world have been concerned about such issues (9–11). Therefore it is safe to assume that in a collection of trial registry records, there will be some errors. Anticipating discrepancies between CTRI and regulatory records is merely an extension of the same assumption that we have with regard to trial records.

Accordingly, we sought to identify the nature of the regulatory letter-CTRI discrepancies and to quantify them. It is important to have transparency around regulatory trials running in India to ensure that the law has been followed and to build public trust in the trial enterprise.

To the best of our knowledge, no other group has performed such a study with Indian data.

## Materials and methods

Here we provide a brief outline of the methodology. Further details are available in Supplementary File S1, and the files referenced therein, that is, Supplementary Files S2–S7, all online. The methodology was referenced (12–18).

The letters providing permission to run particular trials are available as freely accessible pdf files on the regulator’s website (12). A total of 1,000 permission letters were downloaded on 29 May 2023. We wished to extract particular fields of information from these letters, and were able to extract text from about 400 of them. We used an R package ‘pdftools’ to extract text from the downloaded PDFs. Those from which text could not be extracted were often scanned documents. In these cases, problems with features such as the alignment, focus, font, and clarity of the text impeded our ability to extract the data with this package.

To locate the CTRI record corresponding to each letter from the regulator, the title of the study in each letter was compared to the *Scientific Title* of every CTRI record between January 2020 and May 2023. The degree of similarity between two titles was quantified using the Levenshtein distance, a metric for measuring the differences between two sequences of characters based on the number of changes needed to convert the first sequence into the second (17). This was implemented using the ‘RecordLinkage’ function in R (18). Hereafter, we call the Levenshtein distance metric the ‘Similarity score’. The record in the CTRI database with the highest Similarity score was considered the closest, and was paired with the letter from the regulator.

Overall, we obtained a range of Similarity scores (Table 1), and selected the regulatory letter-CTRI pairs where the score was 0.6 or higher. This yielded 304 pairs.

**Table 1:** The distribution of Similarity scores whose values were 0.6 or higher.

Similarity score	Number of pairs	Cumulative number of pairs	Percentage of pairs
0.6–0.69	3	3	1.0
0.7–0.79	13	16	4.3
0.8–0.89	15	31	4.9
0.9–0.99	266	297	87.5
1	7	304	2.3
	304		100

Various fields of data, such as the study title, drug intervention, indication, study sites, and names of the principal investigators, from the regulatory letter and the matching CTRI record, were then entered in paired fashion in an excel file. The data collection and analysis up to this point was carried out by one author. All but one of the subsequent steps were carried out by two authors independently. One of the two authors was always a senior researcher, that is a corresponding author.

**Figure 1:**
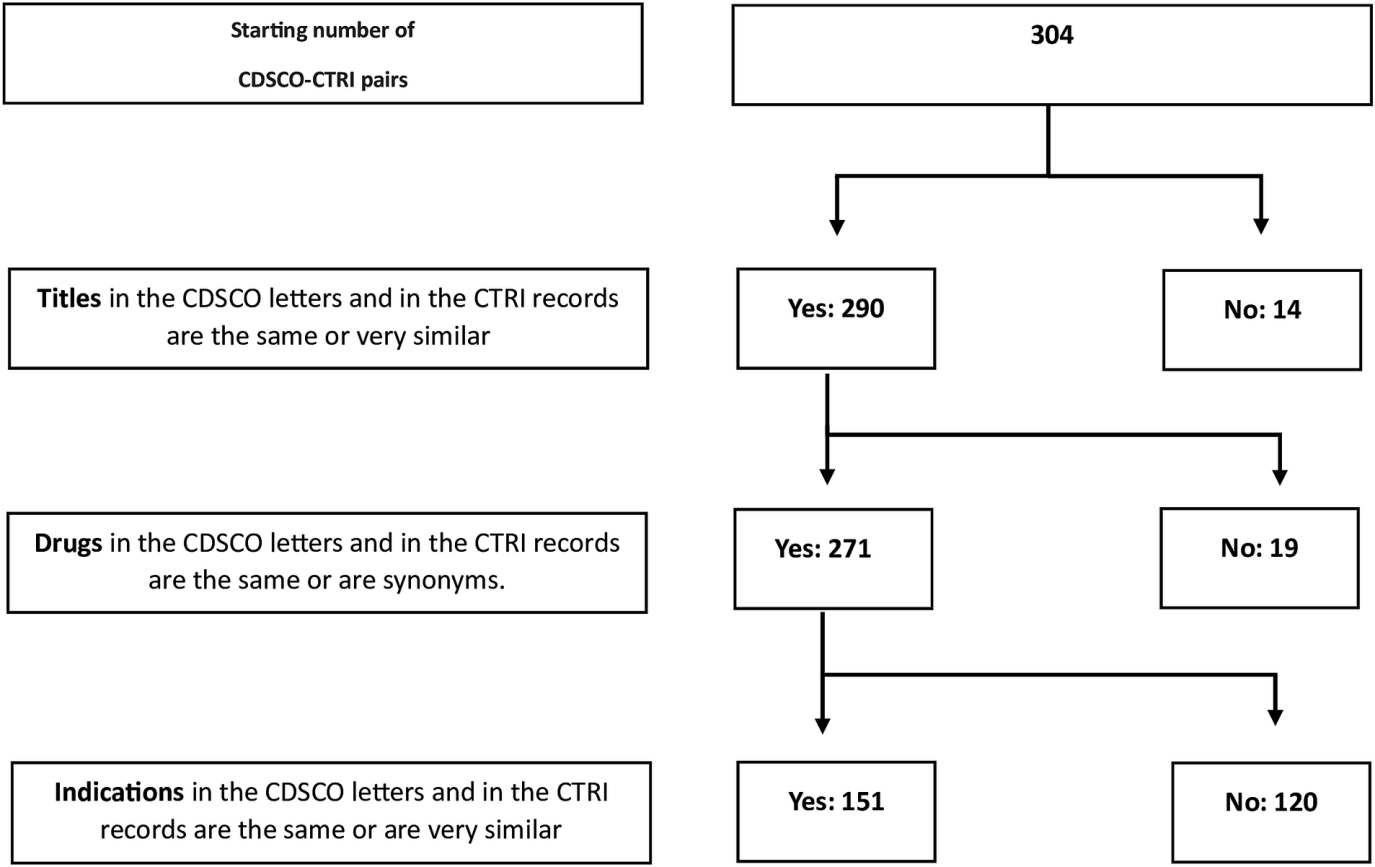
The process of identifying true pairs of letters from the regulator and CTRI records.

After the automated identification of possible regulatory letter-CTRI pairs, we manually checked these 304 pairs to determine whether they were true pairs, that is, whether they were actually the same trial. We did this by successively comparing the titles (Supplementary File S4), the drug interventions (Supplementary File S5), and the indications (Supplementary File S6). The last step was performed by the third author who is a senior medical doctor. After eliminating mismatches, this left us with 151 true pairs (Figure 1).

For each of the 151 pairs, we manually performed the following quantitative assessment. We examined (i) the total number of sites in the regulatory letter versus the total number in the CTRI record; (ii) what fraction of the sites in the letter were present in the CTRI record; (iii) the total number of PIs in the regulatory letter versus the total number in the CTRI record; and (iv) what fraction of the PIs in the letter were present in the CTRI record (Supplementary File S7).

## Results

From the list of sites and PIs of the 151 pairs (Supplementary File S7), we first examined the trial sites. In some cases, the sites matched perfectly; that is, the sites listed in the letter were present in the CTRI record, and there were no additional sites in the latter. However, in other cases, the matches were not perfect. In terms of the number of sites, the pairs could be grouped into three categories: (i) 60 (40%) had the same number of sites, (ii) 79 (52%) had more sites in CTRI, and (iii) 12 (8%) had fewer sites in CTRI. The same situation held true for the number of PIs (Supplementary File S8; Table 2).

**Table 2:** Comparing the number of sites of the trials in 151 letters from the regulator and the corresponding records in CTRI.

Category	Number (percentage)	Comment
The same number of sites in the CTRI records as in the regulatory letters	60 (40)	
More sites in the CTRI record than in the regulatory letter	79 (52)	In 10 cases, the difference in the number of sites was 10 or more. The largest differences were 41, 45 or 58 sites.
Fewer sites in the CTRI record than in the regulatory letter	12 (8)	The difference was a maximum of 5 sites.
TOTAL	151 (100)	

Aside from the difference in the numbers of sites, there were varying degrees of overlap in the identity of the sites between the letters and the CTRI records (Supplementary File S8; Table 3). We expected significant overlap, and found that for 69.5% of the pairs, 100% of the sites listed in the regulatory letter were also found in the CTRI record, suggesting a good overlap. The corresponding figure for the PIs was 58.9% of the pairs. However, there was a wide spread in the percentage of sites (or PIs) listed in the letter that were also found in the CTRI record. Surprisingly, in three pairs there was zero overlap. On re-investigation of these three cases, we found that each letter-CTRI record pair listed the same sponsor, title of the study, intervention, and indication, indicating that they were indeed the same study. In these three zero-overlap cases, (i) one site in the regulatory letter increased to six in CTRI, (ii) five increased to eight and (iii) nine decreased to five.

**Table 3:** The extent to which the specific sites and PIs in the regulatory letter were found in the CTRI record.

Percentage of regulatory letter sites in the CTRI record	Number of cases	Percentage of cases	Percentage of regulatory letter PIs in the CTRI record	Number of cases	Percentage of cases
0	3	2.0	0	3	2.0
1–10	0	0.0	1–10	0	0.0
11–20	4	2.6	11–20	4	2.6
21–30	3	2.0	21–30	5	3.3
31–40	1	0.7	31–40	3	2.0
41–50	5	3.3	41–50	5	3.3
51–60	2	1.3	51–60	6	4.0
61–70	4	2.6	61–70	7	4.6
71–80	14	9.3	71–80	18	11.9
81–90	9	6.0	81–90	10	6.6
91–99	1	0.7	91–99	1	0.7
100	105	69.5	100	89	58.9
Total	151	100		151	100

The following situations were also observed, but very rarely:

- A given letter may have repeated a particular site, but with a different PI. In the CTRI record, these two PIs were affiliated to the original hospital of the letter, and another hospital, respectively.
- A given doctor may have been affiliated to two hospitals in the same city, one listed in the letter and one in the CTRI record.
- A given doctor may have been affiliated to two hospitals in two cities, which could be nearby, more than 150 km apart, or located in very different parts of the country. One of the hospitals was listed in the letter, whereas the other was listed in the CTRI record.

## Discussion

It is widely believed that it is important to have transparency around clinical trials, to ensure that the law has been followed and to build public trust in the trial enterprise (19, 20). Although we are unaware of studies that specifically examine the extent of trust in the Indian trial enterprise, some local trials have been associated with scandals in the past, such as those that resulted in a Parliamentary report (21). As such, we believe that eliminating any errors or discrepancies in any aspect of the trial record ought to contribute to building trust in the Indian trial ecosystem in a proactive way.

As mentioned, the Government of India’s New Drug Rules have been in force since 19 March 2019 (1). According to these rules, a regulatory trial must be registered with CTRI before enrolling any participants. To the best of our knowledge, there are no repercussions if a sponsor does not ensure this registration. The New Drug Rules do not mention any repercussions. Also, we have never come across information pertaining to any such action. In the United States, too, there have hardly been any repercussions for not following the law regarding one particular issue relating to clinical trial records, which we briefly recap here: The Food and Drug Administration Amendment Act came into force in 2016. Thereafter trialists were required to report their results in ClinicalTrials.gov within 12 months of completing the trial. If they did not do so, there was supposed to be a fine of USD10,000 a day. In 2021, it was reported that the rate of reporting results had been so poor that it has been estimated that the United States government could have collected billions of USD in fines. However, it has not done so (22). To return to the Indian scenario, the permission letters from the regulator for the 151 trials of this study were issued from December 2019 onward, and therefore all of these studies were required to be registered with CTRI.

We examined the information related to the sites and PIs, as reflected in the permission letters, and compared it with the information in the CTRI record. In order to do this, we used code that checked the title of the trial as listed in the letter, against the titles of a large number of CTRI records and selected the best match based on a high Similarity score. We chose to use the title for this comparison because earlier research that looked for the same study in different trial registries had found this field to be the most reliable predictor of two records representing the same trial (11, 23).

As such, the first challenge that we had to overcome was matching the regulatory letter with CTRI records. If the CTRI ID was listed in the letter, or the regulatory letter number listed in the CTRI record, this problem would cease to exist. The issue of listing the registry number for a trial in relevant documents of the drug regulator has come up in the United States as well. When the Food and Drug Administration in the United States approves a drug, the publicly available information about the drug does not necessarily include the IDs of the clinical trials that underpinned the approval of the drug (24–26). The former Commissioner of the United States Food and Drug Administration, Dr. Scott Gottlieb, has highlighted the importance of making this connection (27).

Coming back to the regulator-CTRI comparisons, we note that there were several cases of discrepancies regarding the sites and PIs even within the true pairs. Why are the lists discrepant? The regulator issued a circular in 2016 (28) that stated that ‘in the normal course’, the sponsor does not have to apply for a ‘No objection certificate’ for an increase or decrease in sites but should keep the regulator informed of such changes. If the sponsor does not receive an objection from the regulator, then it can go ahead with the change in sites. The New Drug Rules make no mention of the issue of a change of sites (1), and so we presume that the circular still applies. If so, then the lists in CTRI may be the correct ones, since the regulator does not provide any updated information on its own site. Nevertheless, it is known that data in CTRI records may be incomplete or erroneous (6), and so we cannot be sure that the CTRI lists are comprehensive and correct. Furthermore, although occasionally one could understand the origin of the discrepancy, such as when a given doctor was affiliated to two hospitals in the same city, one listed in the letter and one in the CTRI record, we believe that these should have been attended to.

Yet another stakeholder in the process of approving sites and PIs is the Institutional Ethics Committee (hereafter, the Ethics Committee). According to the New Drug Rules, the sponsor has to inform the regulator about Ethics Committee approval for each site, within 15 days of the approval. This implies that the regulator is definitely in the know of changes in sites for a given study. However, as mentioned previously, the New Drug Rules do not require that the regulator approve (or even acknowledge the proposal for) changes in the number of sites after initial regulatory approval of the trial. Just as there is value in making public the regulator’s initial approval of a trial, with details of the approved sites and PIs, we believe that there is value in making public any revisions in these lists. If that is not done, then the information on the letter and in the CTRI record will be discrepant. First, one is not sure which list is correct and to what extent. Second, in such a situation we do not know whether the sponsor had actually informed the regulator of the changes. If not, this would be in breach of the law. And third, to the extent that a publicly-available letter from the regulator helps to build confidence in the regulatory process, the lack of consistent information undermines this confidence. As has been pointed out by a committee set up by the House of Commons of the United Kingdom (19), transparency around trials increases the public’s trust in the trial ecosystem. A lack of trust could lead to a societal backlash as has been seen in many people losing faith in vaccines.

The Ethics Committee can also play a role in reducing the above-mentioned discrepancies. According to current norms (29), the process for regulatory and Ethics Committee approvals can run in parallel, without the need for one to wait for formal approval by the other. We believe that at each site, at the time of issuing the approval letter, the Ethics Committee should insist on receiving copies of CTRI registration and of regulatory approval, as and when these are received by the sponsor. For an additional trial site being roped in, the Ethics Committee at that site should also insist on receiving proof of the newly added trial site in the CTRI record. In addition, the Ethics Committee must independently communicate its approval to the regulator, irrespective of whether or not the sponsor does so. Finally, we believe that the regulator should make public the revised lists of sites and PIs in the same manner as it does the originally approved lists.

This study had some limitations. First, for a large number of regulatory letters, extracting data was an insurmountable challenge. Therefore, the sample size for this study was smaller than it needed to be. We are unsure if the results we obtained can be extrapolated to other letters. Second, although we used title, drug and indication to find CTRI matches for the trials listed in the regulatory documents, some pairs may still not have been identified correctly. This means that we may have had a few false positives (where a pair was identified as a true pair, although it was not). The only way this could have happened was if two trials had titles, drug interventions and indications so similar that a senior medical doctor could not differentiate between them. We have no reason to believe that there were a large number of false positives, and hence assume that their numbers were small, with minimal impact on our conclusions. Alternatively, we may have had a few false negatives (where a true pair was not identified, although such a pair existed). Since we ran the title of the regulatory letter against thousands of CTRI records, and provided a wide window (0.6 Similarity score or above), any match that we have missed must have drastically different titles in the letter and CTRI record. This is highly unlikely. Any such missed pairs would have contributed to a slightly larger dataset. Again, we believe that this would not have impacted our results significantly.

In summary, we evaluated 151 letters from the drug regulator that gave permission to run particular clinical trials, and the CTRI records that matched those trials. In these regulatory letter-CTRI paired records, we examined the details of the study sites and associated PIs. Based on the challenges that we faced, it is clear that (i) the regulatory letters need to be easier to extract data from and (ii) the regulatory letters and CTRI records need to cross-reference each other. Further, (iii) the regulator should make public any changes made to the letter that grants permission for a trial, to increase transparency and confidence in the regulatory process for approving trials in India, and (iv) the Ethics Committees at a new site should insist on receiving proof of the newly added trial site in the CTRI registration. These steps will increase transparency around regulatory trials running in India, and will improve the accuracy of the records of such studies.

## Data availability statement

The original contributions presented in the study are included in the article/Supplementary material, further inquiries can be directed to the corresponding author.

## Author contributions

IB: Data curation, Formal analysis, Methodology, Software, Writing – original draft, Writing – review & editing. AR: Data curation, Formal analysis, Validation, Visualization, Writing – review & editing. RV: Data curation, Formal analysis, Methodology, Writing – review & editing. GS: Conceptualization, Formal analysis, Funding acquisition, Methodology, Project administration, Validation, Visualization, Writing – original draft, Writing – review & editing.
