# Supplementary material for: Data from the Indian drug regulator and from Clinical Trials Registry-India does not always match
Source: Front Med (Lausanne). 2024 Feb 15;11:1346208. doi: 10.3389/fmed.2024.1346208 (PMC10906088; doi:10.3389/fmed.2024.1346208)
Supplement: Supplementary file 1 [file Data_Sheet_1.doc]

**Supplementary File S1. Expanded methodology**

**Note:** All references are detailed in the main text.

The letters providing permission to run particular trials are available as pdf files from CDSCO (12). These letters pertain to the following categories of interventions: GCT (global clinical trial, ie a multinational one), rDNA (recombinant DNA), SND (subsequent new drug), New drug, FDC (fixed dose combination), Vaccine, and IND (investigational new drug). We included all of these categories in this study.

**The CDSCO permission letters**

The permission letters from the CDSCO database were downloaded on 29 May 2023. The text from these permission letters was extracted using the extract_text() function from the ‘pdftools’ package (13) in R, version 4.1.2 (14) which is available in Supplementary File S2.R. A total of 1000 permission letters were downloaded, and we could extract text from approximately 400 of them.

Once extracted, the text from each CDSCO letter was submitted as a prompt to ChatGPT using the package ‘openai’ (15) in Python version 3.1 (16). The specific language processing model used was ‘gpt-3.5-turbo’, with the ‘temperature’ parameter set to 0.17, a deliberately low value to limit the randomness of the response.

Accompanying the text were the following questions. They were preceded by the line ‘Answer the following questions with information only present in the prompt’.’:

1. What is the name and full address of the person who has been granted permission to conduct this trial?
2. What is the full address of the person that this letter is addressed to?
3. What is the title of the clinical trial?
4. What is the place and date of the document?
5. What is the protocol number, including version and date, of the trial?
6. What is the application reference number, which can be called a file number and could start with ‘BIO/CT’ or ‘GCT’, including the date on which the application was submitted?
7. In a numbered list, provide the names of the drug.
8. What are the therapeutic classes of the new drug?
9. What are the dosage forms of the new drug?
10. What is the composition of the new drug, differentiated in the form of a list into active and inactive ingredients if possible.
11. What are the indications of the new drug?
12. List the details of the clinical trial sites, including, in the form of a numbered list, the names and address, the ethics committee details, ECR number beginning with ‘ECR’, and the name of the investigator.

Questions 1 and 2 referred to the same field of data, which could be present in different forms in the CDSCO letter, requiring two differently worded questions to extract the data.

**The CTRI records**

Between 27–29 May 2023, we downloaded certain fields of information (page number, CTRI number, public title and scientific title) for all the CTRI records registered on dates back to 1 January 2020. The page number means the number added to the URL, as explained here: We accessed the CTRI site at ‘http://ctri.nic.in/Clinicaltrials/pmaindet2.php?trialid=’ and downloaded all the records by adding numbers (1 to several hundreds of thousands) to the end of the URL in order to capture all records.

**Finding CDSCO-CTRI matches**

To locate the CTRI record corresponding to each CDSCO letter, the title of the trial in each CDSCO letter was compared to the *Scientific Title* field of every CTRI record registered between January 2020 and May 2023. The degree of similarity between two titles was quantified using the Levenshtein distance (17), implemented using the ‘RecordLinkage’ function in R (18). In this work, we call the Levenshtein distance metric ‘the Similarity score’. The record in the CTRI database with the highest Similarity score was considered the closest, and was paired with the CDSCO pdf.

Having thus identified the appropriate record in the CTRI database, the remaining data points, namely the registration date, drug name, indication, intervention, details of the clinical trial sites, and names of the principal investigators, were extracted from the relevant CTRI page using custom built code in R which is available in Supplementary File S3.R.

We selected CDSCO-CTRI pairs where comparison of the trial titles produced a Similarity score of 0.6 or higher. This threshold was determined by manual assessment of titles between CDSCO-CTRI pairs. The score was high enough to eliminate mismatches, but low enough to accommodate minor differences in phrasing between the titles in the CDSCO and CTRI records.

Various fields of data (Title, Drug name, Indication, Sites, PIs) from the CDSCO pdf and the matching CTRI record were then entered, in paired fashion in an excel file.

After the automated identification of possible CDSCO-CTRI pairs, another author manually crosschecked the data extracted from each CDSCO pdf with the data in the spreadsheet and corrected any errors.

The work up to this point was done by one author. All but one of the subsequent steps were carried out by two authors, independently. One of the two authors was always a senior researcher, that is a corresponding author. Discrepancies were resolved through discussion.

We then manually checked these 304 pairs to determine whether they were ‘true pairs’, that is whether they were actually the same trial. We did this by successively comparing the titles (details in Supplementary File S4), the drug interventions (Supplementary File S5) and the indications (Supplementary File S6). The last step was performed by a senior medical doctor. After eliminating mis-matches, this left us 151 true pairs. For each of the 151 pairs, we determined (i) the total number of sites in the CDSCO letter versus the total number in the CTRI record; (ii) what fraction of the CDSCO sites were present in the CTRI record; (iii) the total number of PIs in the CDSCO letter versus the total number in the CTRI record; and (iv) what fraction of the CDSCO PIs were present in the CTRI record. This data is available in Supplementary File S7.
