# Supplementary material for: Data from the Indian drug regulator and from Clinical Trials Registry-India does not always match
Source: Front Med (Lausanne). 2024 Feb 15;11:1346208. doi: 10.3389/fmed.2024.1346208 (PMC10906088; doi:10.3389/fmed.2024.1346208)
Supplement: Supplementary file 2 [file Data_Sheet_2.docx]

library(pdftools)

library(stringr)

library(writexl)

library(data.table)

library(rvest)

library(RCurl)

rm(list = ls())

#Setting the location of the downloaded CDSCO permission letters, which are saved in

# a .pdf format.

setwd('') #Insert location of files

file_list = list.files(getwd())

#Extracting the text from all the downloaded files and saving the extracted text

# as .txt files.

for(i in 1:length(file_list))

{

#Selecting individual files in the folder.

input_path = file_list[i]

#Identifying the number of pages in the document.

total_pages = pdf_info(input_path)$pages

page_numbers = seq(1, total_pages, 1)

all_pages = extract_text(input_path, pages = page_numbers)

#Identifying the different sections in the document. There are usually pages in the middle

# that contain no useful information. Text from these pages are not extracted.

formct_start= which(grepl('FORM CT', all_pages, fixed = TRUE))

annex_start= which(grepl('Annexure:', all_pages, fixed = TRUE))

if(length(formct_start) > 0 | length(annex_start) > 0)

{

mid_point = min(annex_start, formct_start)

#Extracting text from the page containing the text 'FORM CT'

first_half = extract_text(input_path, pages = c(1))

#Extracting text from the pages including and following the page

# labelled 'Annexure'

second_half = extract_text(input_path, pages = seq(mid_point, total_pages, 1))

combined = c(first_half, second_half)

#Writing the useful text to a .txt file in the appropriate folder.

write.table(combined, paste('../', input_path, '.txt'), sep = '')

}

}
