# Supplementary material for: Data from the Indian drug regulator and from Clinical Trials Registry-India does not always match
Source: Front Med (Lausanne). 2024 Feb 15;11:1346208. doi: 10.3389/fmed.2024.1346208 (PMC10906088; doi:10.3389/fmed.2024.1346208)
Supplement: Supplementary file 3 [file Data_Sheet_3.docx]

library(tidyverse)

library(tabulizer)

library(pdftools)

library(dplyr)

library(stringr)

library(writexl)

library(data.table)

library(rvest)

library(xml2)

library(XML)

library(RCurl)

library(future)

library(RecordLinkage)

rm(list = ls())

#Here I select those CTRI pages that are present in my list of 300 pdfs, having previously

# identified their CTRI page numbers. Using these page numbers, I navigate to the #webpage and extract relevant information, namely: Indicatin, Intervention, Drug name, Registration #date, and Site data.

output_data = data.frame()

#Here I read the file containing the page numbers of CTRI trials where the 'Trial title'

# matched the 'Scientific title' field of a CDSCO trial that is being considered for this

# analysis.

page_numbers = read.csv('')

for (i in 1:length(page_numbers))

{

#I delete any values being stored in the variables that will be saved at the end of this #iteration.

CTRI_Page_number = NULL

CTRI_Indication = NULL

CTRI_Intervention = NULL

CTRI_Drug_name = NULL

CTRI_Registration_date = NULL

CTRI_Sites_data = NULL

print(paste('Index ', i), sep = '')

print(paste('Page ', page_numbers[i]), sep = '')

CTRI_Page_number = page_numbers[i]

#I access the CTRI database and the page number of interest

myurl = paste0("http://ctri.nic.in/Clinicaltrials/pmaindet2.php?trialid=", CTRI_Page_number, sep = '')

url = url(paste0("http://ctri.nic.in/Clinicaltrials/pmaindet2.php?trialid=",CTRI_Page_number, sep = ''))

ctri_page = read_html(url)

page_data = ctri_page %>% html_elements('td') %>% html_text2()

test_table = ctri_page %>% html_table()

largest_table = max(sapply(test_table[][1], dim))

table_selected = data.frame(test_table[lengths(test_table)== largest_table])

table_selected = table_selected[ 2:nrow(table_selected) , ]

#I identify the location of the Indication field, stored in the website under the

# title 'Health Condition / Problem Studied'

Indications_location = grep('Health Condition / Problems Studied', table_selected$X1)

CTRI_Indication = table_selected$X2[Indications_location+2]

#I identify the location of the 'Intervention' field

Intervention_location = grep('Intervention', table_selected$X1)[2]

CTRI_Intervention = table_selected$X3[Intervention_location]

Drug_name_location = grep('Intervention', table_selected$X1)[2]

CTRI_Drug_name = table_selected$X2[Drug_name_location]

#Identifying the row number of the clinical sites:

Sites_location = grep('Name of Principal', table_selected$X1)

#Identify the rownumber of the ethics committee details.

ethics_committee_location = grep('Details of Ethics Committee', table_selected$X1)

#Seleting the appropriate rows for the site locations

CTRI_Sites = table_selected[((Sites_location+1):(ethics_committee_location-1)), c('X1', 'X2', 'X3') ]

#Comparing the two columns to see if there are repeats, and if so, deleting the repeats

CTRI_Sites$X2 = gsub('\r\n', '', CTRI_Sites$X2)

CTRI_Sites$X2 = gsub(' ', ' ', CTRI_Sites$X2)

CTRI_Sites$X3 = gsub('\r\n', ' ', CTRI_Sites$X3)

CTRI_Sites$X2[CTRI_Sites$X2 == substr(CTRI_Sites$X3, 1, nchar(CTRI_Sites$X2))] = ''

CTRI_Sites$X2 = paste('Site', ((as.numeric(rownames(CTRI_Sites)) + 1) - as.numeric(rownames(CTRI_Sites)[1])),

':', CTRI_Sites$X2, CTRI_Sites$X3, sep = ' ')

CTRI_Sites$X1 = paste('Name of principal investigator: ', CTRI_Sites$X1)

#Saving it all into one column.

CTRI_Sites$Sites = paste(CTRI_Sites$X2, CTRI_Sites$X1, sep = '. ')

CTRI_Sites_data = paste0(CTRI_Sites$Sites, collapse = '. ')

Registration_date = table_selected$X2[2]

Registration_date_start = unlist(gregexpr('on:',Registration_date))

Registration_date_end = unlist(gregexpr(']',Registration_date))

CTRI_Registration_date = substr(Registration_date, (Registration_date_start + 4), (Registration_date_end-1))

#Saving all the relevant data into a single row, to be added to the end of the master #dataframe containing the information from all CTRI pages.

tmp_row = data.frame(CTRI_Page_number, CTRI_Indication, CTRI_Intervention, CTRI_Drug_name, CTRI_Registration_date, CTRI_Sites_data)

output_data = rbind(output_data, tmp_row)

}
